# Vegan gummy candies with low calorie based on celery (*Apium graveolens*) puree and boswellia gum (*Boswellia thurifera*)

**DOI:** 10.1002/fsn3.4190

**Published:** 2024-05-22

**Authors:** Saba Ghodsi, Marjan Nouri

**Affiliations:** ^1^ Department of Food Science and Technology Roudehen Branch, Islamic Azad University Roudehen Iran

**Keywords:** central composite design, functional property, gummy candy, optimization

## Abstract

Gummy candy is one of the main snacks for children, and conventional samples with high calorie illustrate no nutritional value; therefore, the aim of present research was to develop functional product on priority. Celery (*Apium graveolens*) puree (25%–50%), boswellia gum (10%–20%), lemon essential oil (0.25%–0.50%), and sugar (10%–20%) in two levels were considered for vegan gummy candy production. Based on central composite design, the 30 types of gummy candies were prepared; afterward, response surface methodology was applied to optimize results determined by texture (hardness, springiness, adhesiveness, gumminess, chewiness, and elasticity characteristics), physicochemical attributes (pH, sugar content, water activity, antioxidant function, and calorie restriction), and also sensory evaluation. In general, elevated concentration of celery puree and boswellia gum‐enhanced hardness, chewiness, and also gumminess for treated products. On the other hand, higher sugar with lemon essential oil improved adhesion, springiness, and elasticity features. More boswellia gum, celery, lemon essential oil, and reduction in sugar elevated water activity and also declined pH for treated samples. The celery puree, boswellia gum, and lemon essential oil significantly enhanced antioxidant function of treated gummy candies. According to attained results, sugar had a remarkable influence on acceptability and in treated samples calorie decreased. Based on all investigated factors, optimal formulation was achieved including 25% celery puree, 20% boswellia gum, 0.450% lemon essential oil, and 13.55% sugar. Regarding the results, obtained gummy candy with high nutritional value and low calorie demonstrated the potential to produce extensively in food sector.

## Graphical abstract

Gummy candy is one of the main snacks for children, and conventional samples with high calorie illustrate no nutritional value; therefore, the aim of present research is to develop functional product on priority. Celery puree (25%–50%), boswellia gum (10%–20%), lemon essential oil (0.25%–0.50%), and sugar (10%–20%) in two levels were considered for vegan gummy candy production.

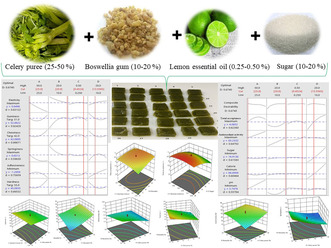

## 1 INTRODUCTION

Nowadays, gummy candies are one of the snacks with firm consistency, elastic texture, and shiny appearance, which have long been liked by a wide range of consumers, especially children (Paternina et al., 2022). The main ingredients are gelling agents, sweeteners, acids, essential oils, and food pigments for producing gummy candies (Moghaddas Kia et al., 2020; Uribe‐Wandurraga et al., 2021).

The nutritional and biological potential for vegetables such as lack of cholesterol and high nutrients including vitamins, antioxidants, and minerals has caused multiple attributes in preparation of various products (Vatankhah Lotfabadi et al., 2020). Celery (*Apium graveolens*) contains vitamins (A, C, B_9_, B_1_, B_5_, B_6_, and E), minerals (calcium, iron, copper, potassium, magnesium, phosphorus, manganese, and sodium), microelements (Fe, Mn, B, Zn, and Cu), and also amino acids (valine, isoleucine, tryptophan, and aspartic acid), which has a high nutritional and biological value (Babalar et al., 2023; Mohsenpour et al., 2023). The influences of celery powder on bread quality produced with wheat flour had been investigated (Wang et al., 2020).

Citrus lemon includes volatile essential oil, which is obtained by pressing outer skin of fresh fruit (Oikeh et al., 2016; Yazgan et al., 2019). It is cultivated for fresh consumption but according to various phenolic and flavonoid contents has become extremely prominent in food and pharmaceutical industries (Shafaghi Rad & Nouri, 2023). Lemon essential oil consists of distinct components such as aliphatic sesquiterpenes, terpenes, oxygenated derivatives, and aromatic hydrocarbons with medicinal, antimicrobial, antifungal, antioxidants, and anticancer features (Makni et al., 2018).

Hydrocolloids such as gums are natural origin polysaccharides in a variety of forms including seed, mucilage, and exudate, which frequently applied as thickeners, stabilizers, emulsifiers, clarifying, and gelling agents in food industry (Razjoo et al., 2021; Tireki et al., 2023). Boswellia gum is obtained from *Boswellia* genus of the *Burseraceae* family, and these species produce oleoresin gum, which are traditionally harvested by making incisions on trunks of trees (Jameel & Mohammed, 2021; Miran et al., 2022). Boswellia resin is available in yellowish, blue, and green colors including 27%–35% gum, which lipophilic part is a rich source of terpenoids, especially boswellic acids as the important medicinal group with antioxidant and anticancer attributes (Almeida‐Da‐Silva et al., 2022; Gupta et al., 2022). In addition to technological application in gel formation, this gum demonstrated significant nutritional function that was applied in film composite production with chitosan (Jameel & Mohammed, 2021), silver nanoparticles (Kora et al., 2012), and nanocellulose derived from sugarcane bagasse (Reshmy et al., 2021). Texture, shelf life, and microstructure of gummy candies are highly dependent on processing, type and polymer proportion, and sweeteners in formulation (Zhang & Barringer, 2018).

In previous researches, gummy candy preparation had been evaluated using different glucose, its syrup, sucrose, starch or gelatin (Tireki et al., 2023), *Spirulina* and freeze‐dried açai pulp (Paternina et al., 2022), molasses types and gelatin ratios (Kurt et al., 2022), ciku (*Manilkara zapota*) fruit (Hashim et al., 2021), licorice plant with agar (Basiri, 2020), encapsulated d‐limonene (Vatankhah Lotfabadi et al., 2020), gelatin and Arabic gum (Zainol et al., 2020), red beet extract, *Salix aegyptiaca* distillate, and gellan gum (Moghaddas Kia et al., 2020), and also gelatin, pectin, and starch (Zhang & Barringer, 2018).

The aim of the present research was to produce functional vegan gummy candies; therefore, celery puree (25%–50%), boswellia gum (10%–20%), lemon essential oil (0.25%–0.50%), and sugar (10%–20%) were applied in two levels and based on central composite design (CCD), the 30 types of gummy candy samples were produced; then, response surface methodology (RSM) was used to optimize results determined by texture (hardness, springiness, adhesiveness, gumminess, chewiness, and elasticity characteristics), physicochemical attributes (pH, sugar content, water activity, antioxidant function, and calorie restriction), and also sensory evaluation.

## 2 MATERIALS AND METHODS

### 2.1 Chemical components

The obtained celery (*Apium graveolens*) plant, boswellia seed, and lemon fruit were purchased from a local market available. The 2,2‐diphenyl‐1‐picrylhydrazyl (DPPH), Fehling's solution, and zinc acetate were attained from Merck, Germany, and also other chemicals and reagents were of analytical grade.

### 2.2 Preparation of celery puree

Fresh celery was procured from the local market; after removing impurities, it was completely washed, drained, and then crushed in a blender, and a smooth puree was prepared. Finally, the puree produced from the sieve was smoothed with 10 meshes to make completely homogeneous and uniform structure (Wang et al., 2020).

### 2.3 Manufacture of boswellia gum

Boswellia seeds were powdered using a mortar; so, 25 g level was mixed with 200 mL deionized water and stirred at a speed of 800 rpm for 24 h. The obtained mixture was separated at 1500 rpm during 10 min; after removing impurities, the resulted liquid was again centrifuged at 2500 rpm and successively 10,000 rpm about 10 and 20 min, respectively. At last, the attained gum was separated with a net and dried through a freeze‐dryer (DG‐65Z04‐10A, China) for 24 h (Namdarian et al., 2018).

### 2.4 Essential oil extraction from lemon

The 20 g of washed lemon was placed in a distillation flask and after filling with 1 to 5 ratio (w/v: lemon to water), the essential oil was prepared using Clevenger‐type apparatus for 4 h. The obtained essential oil was entirely dried using sodium sulfate and stored in a glass container at 4°C temperature until the test (Yazgan et al., 2019).

### 2.5 Optimization of gummy candy preparation based on celery puree

To prepare gum candy, boswellia powder was produced by diluting it in water at a ratio of 1:70. Gelatin was normally insoluble in water and therefore, dissolved in hot water (less than 50°C) before being added to mixture. Then, celery puree (25%–50%), boswellia gum (10%–20%), sugar (10%–20%), lemon essential oil (0.25%–0.5%), and lecithin E471 (0.1%) emulsifier were added to mixture. The resulted samples were cooked for 10 min (Brix value set to 60°C), and citric acid was added to cooled mixture (Table 1) and also based on CCD, 30 types were prepared with different concentrations. The prepared blend was poured into silicone containers with dimensions of 1.9 cm × 1.9 cm × 1.9 cm, which were placed in refrigerator at 4°C for 24 h. The gels were removed from prepared blends, and prepared gummy candies were stored in plastic packaging in refrigerator to perform tests (Teixeira‐Lemos et al., 2021).

**TABLE 1:** Independent variables and their levels used in central composite design for gummy candy samples.

Factors	Coded symbols	Levels	
		−1	1
Celery puree (%)	A	25	50
Boswellia gum (%)	B	10	20
Lemon essential oil (%)	C	0.25	0.5
Sugar (%)	D	10	20

### 2.6 Comparative tests on gummy candy samples

#### 2.6.1 Texture analysis

The hardness, springiness, adhesiveness, gumminess chewiness, and elasticity characteristics of stored samples in containers (35 mm diameter and 20 mm height) were measured by a texture analyzer (Stable Micro System, TA. HD Plus, Godalming, UK). For each sample, three measurements with three replicates were performed using a cylindrical probe (P/36R). The applied time between two compressions (50%) to samples was set to 2S (Kurt et al., 2022).

#### 2.6.2 Physicochemical determination

The gummy candy (5 g) was mixed and homogenized in distilled water (20 mL); then, pH was calculated with a pH meter (Metrohm 827, Switzerland). The sugar level of gummy candy samples was determined by Linn‐Ainon method using Fehling's solution (Piazza et al., 2009). The sample (2 g) was ground, placed in a disposable cup, and analyzed inside chamber of water activity measuring device (Meter Aqualab 4TE, Pullman, WA) at room temperature (Tireki et al., 2023).

#### 2.6.3 Evaluation of antioxidant attribute

Initially, 0.2 mL prepared samples were added to 4 mL methanol solution of 60 μM DPPH free radical and kept for 1 h at room temperature in dark. Afterward, the sample absorbance was read and reported at 517 nm wavelength using a spectrophotometer (Wang et al., 2020).

#### 2.6.4 Calorie restriction

The gummy candy samples were dried overnight in an oven to remove all the moisture, and standards were measured before calibrating bomb calorimeter (IKA C2000, USA). Approximately, 1 g sample was weighed after drying into a special container and exactly 2 L water was placed inside the well of bomb bucket calorimeter. The adjustment system and device were turned on, and also calorie content was measured in terms of kcal (Azuan et al., 2020).

### 2.7 Sensory evaluation

The sensory assessment for samples including total acceptance with 5‐point hedonic method (at evaluation levels 1 to 5; 1: unacceptable or extremely poor; 2: unacceptable or poor; 3: acceptable or average; 4: satisfactory or good; and 5: extremely satisfactory or good) was done by 40 panelists (20 women and 20 men). The evaluation was conducted in individual sensory booths consistent with ISO 8589 acceptable method (Shafaghi Rad & Nouri, 2023).

### 2.8 Statistical approach

In the present research, celery puree (25%–50%), boswellia gum (10%–20%), sugar (10%–20%), and lemon essential oil (0.25%–0.50%) were optimized for manufacturing gummy candy. According to CCD, 30 types of samples were produced; then, RSM was used to optimize results measured through texture (hardness, springiness, adhesiveness, gumminess, chewiness, and elasticity functions), physicochemical features (pH, sugar, water activity, antioxidant, and calorie restriction), and also sensory assessment. All computation and graphics were performed using statistical software Design Expert v.13 and Microsoft Excel 2020. All measurements were repeated at least twice using freshly prepared samples, and values were expressed as means ± standard deviation (SD).

## 3 RESULTS AND DISCUSSION

### 3.1 Results related to texture evaluation of gummy candy samples through RSM

Texture is the result of complex arrangement for various interactions between constituents and structural elements at both macroscopic and microscopic scales (Azmoon et al., 2021). Hardness, adhesiveness, springiness, chewiness, gumminess, and elasticity are the most important texture descriptors for gel sweets (Hashim et al., 2021). The actual and predicted results for statistical analysis of gummy candy samples based on CCD in 30 test steps are presented, and variable optimization is done using RSM in Tables 2 and 3.

**TABLE 2:** Central composite design with actual and predicted values of hardness, adhesiveness, and springiness responses based on independent variables.

Run	Independent variables (%)				Dependent variables (responses)					
	A	B	C	D	Hardness (N)		Adhesiveness (N)		Springiness (−)	
					Actual value	Predicted value	Actual value	Predicted value	Actual value	Predicted value
1	25.0	10	0.250	10	39.02	35.88	−1.25	−1.21	0.87	0.85
2	50.0	10	0.250	10	67.40	71.49	−1.35	−1.39	0.45	0.47
3	25.0	20	0.250	10	56.40	57.71	−1.30	−1.34	0.43	0.47
4	50.0	20	0.250	10	81.64	82.35	−1.40	−1.36	0.29	0.23
5	25.0	10	0.500	10	32.39	33.87	−1.22	−1.18	0.75	0.80
6	50.0	10	0.500	10	65.12	67.61	−1.32	−1.36	0.43	0.44
7	25.0	20	0.500	10	53.98	52.99	−1.28	−1.31	0.46	0.47
8	50.0	20	0.500	10	75.09	75.77	−1.36	−1.33	0.32	0.25
9	25.0	10	0.250	20	27.23	27.40	−0.95	−0.98	0.88	0.98
10	50.0	10	0.250	20	61.78	61.39	−1.32	−1.28	0.62	0.58
11	25.0	20	0.250	20	51.53	47.66	−1.28	−1.22	0.58	0.55
12	50.0	20	0.250	20	71.30	70.68	−1.31	−1.35	0.30	0.29
13	25.0	10	0.500	20	25.40	23.32	−0.91	−0.94	0.91	0.94
14	50.0	10	0.500	20	55.90	55.45	−1.28	−1.24	0.57	0.57
15	25.0	20	0.500	20	44.10	40.87	−1.23	−1.18	0.56	0.57
16	50.0	20	0.500	20	60.28	62.04	−1.29	−1.32	0.34	0.33
17	25.0	15	0.375	15	37.58	47.94	−1.21	−1.22	0.95	0.72
18	50.0	15	0.375	15	84.60	76.32	−1.42	−1.38	0.29	0.41
19	37.5	10	0.375	15	36.41	34.24	−1.21	−1.18	0.91	0.72
20	37.5	20	0.375	15	44.20	48.45	−1.29	−1.29	0.33	0.41
21	37.5	15	0.250	15	42.07	43.81	−1.24	−1.22	0.58	0.54
22	37.5	15	0.500	15	38.14	38.48	−1.20	−1.19	0.61	0.53
23	37.5	15	0.375	10	75.05	68.42	−1.37	−1.32	0.21	0.19
24	37.5	15	0.375	20	48.60	57.31	−1.18	−1.20	0.38	0.29
25	37.5	15	0.375	15	54.60	51.11	−1.23	−1.25	0.49	0.45
26	37.5	15	0.375	15	45.30	51.11	−1.26	−1.25	0.45	0.45
27	37.5	15	0.375	15	52.70	51.11	−1.24	−1.25	0.31	0.45
28	37.5	15	0.375	15	54.09	51.11	−1.27	−1.25	0.31	0.45
29	37.5	15	0.375	15	54.20	51.11	−1.29	−1.25	0.41	0.45
30	37.5	15	0.375	15	52.02	51.11	−1.20	−1.25	0.45	0.45

**TABLE 3:** Central composite design with actual and predicted values of chewiness, gumminess, and elasticity responses based on independent variables.

Run	Independent variables (%)				Dependent variables (responses)					
	A	B	C	D	Chewiness (N)		Gumminess (N)		Elasticity (−)	
					Actual value	Predicted value	Actual value	Predicted value	Actual value	Predicted value
1	25.0	10	0.250	10	31.12	25.87	38.34	34.71	0.67	0.68
2	50.0	10	0.250	10	47.52	52.16	53.39	58.54	0.56	0.53
3	25.0	20	0.250	10	45.34	47.80	56.54	58.45	0.54	0.54
4	50.0	20	0.250	10	62.32	62.21	69.94	67.52	0.36	0.32
5	25.0	10	0.500	10	23.81	27.63	37.89	39.20	0.71	0.74
6	50.0	10	0.500	10	55.79	53.07	60.63	59.37	0.56	0.52
7	25.0	20	0.500	10	54.34	48.31	65.13	60.23	0.58	0.55
8	50.0	20	0.500	10	58.34	61.88	61.09	65.65	0.29	0.27
9	25.0	10	0.250	20	21.09	19.44	27.96	24.67	0.75	0.77
10	50.0	10	0.250	20	45.98	47.99	52.51	54.09	0.63	0.64
11	25.0	20	0.250	20	43.65	42.35	54.34	52.28	0.6	0.62
12	50.0	20	0.250	20	60.97	59.04	66.98	66.94	0.45	0.42
13	25.0	10	0.500	20	19.81	15.90	23.93	23.03	0.85	0.87
14	50.0	10	0.500	20	44.18	43.61	49.44	48.80	0.67	0.67
15	25.0	20	0.500	20	40.33	37.57	51.82	47.94	0.66	0.68
16	50.0	20	0.500	20	52.17	53.41	58.63	58.94	0.43	0.41
17	25.0	15	0.375	15	27.46	42.07	35.76	51.19	0.86	0.73
18	50.0	15	0.375	15	69.23	63.13	75.86	68.61	0.4	0.52
19	37.5	10	0.375	15	24.15	27.78	30.65	32.32	0.79	0.73
20	37.5	20	0.375	15	38.76	43.64	42.76	49.27	0.47	0.52
21	37.5	15	0.250	15	35.75	36.88	40.81	43.59	0.55	0.55
22	37.5	15	0.500	15	27.56	34.94	36.43	41.83	0.58	0.57
23	37.5	15	0.375	10	57.62	57.26	63.61	62.89	0.44	0.52
24	37.5	15	0.375	20	40.93	49.80	45.62	54.52	0.72	0.63
25	37.5	15	0.375	15	42.31	44.70	50.14	50.28	0.58	0.60
26	37.5	15	0.375	15	46.82	44.70	51.02	50.28	0.61	0.60
27	37.5	15	0.375	15	51.56	44.70	55.59	50.28	0.59	0.60
28	37.5	15	0.375	15	54.97	44.70	54.98	50.28	0.63	0.60
29	37.5	15	0.375	15	48.09	44.70	59.25	50.28	0.58	0.60
30	37.5	15	0.375	15	49.98	44.70	55.24	50.28	0.65	0.60

Hardness is the force required to compress sample between molars or achieve a specific deformation. Based on achieved results (Table 2), the highest hardness of gummy candy was distinguished in run number 4 (81.64 N) and the lowest level 13 (25.4 N); therefore, it was obtained to predict this feature (Equation 1):

(1) $$\begin{array}{c} \text{Hardness}=52.15+12.65A+5.61B-2.12C–5.23D–2.80\mathrm{AB}\\ \;–0.40\mathrm{AC}–0.73\mathrm{BC}–0.45\mathrm{BD}–0.45\mathrm{CD}+2.82A^{2}\\ \;–2.38\;B^{2}–2.43C^{2}+3D^{2} \end{array}$$

**FIGURE 1:**
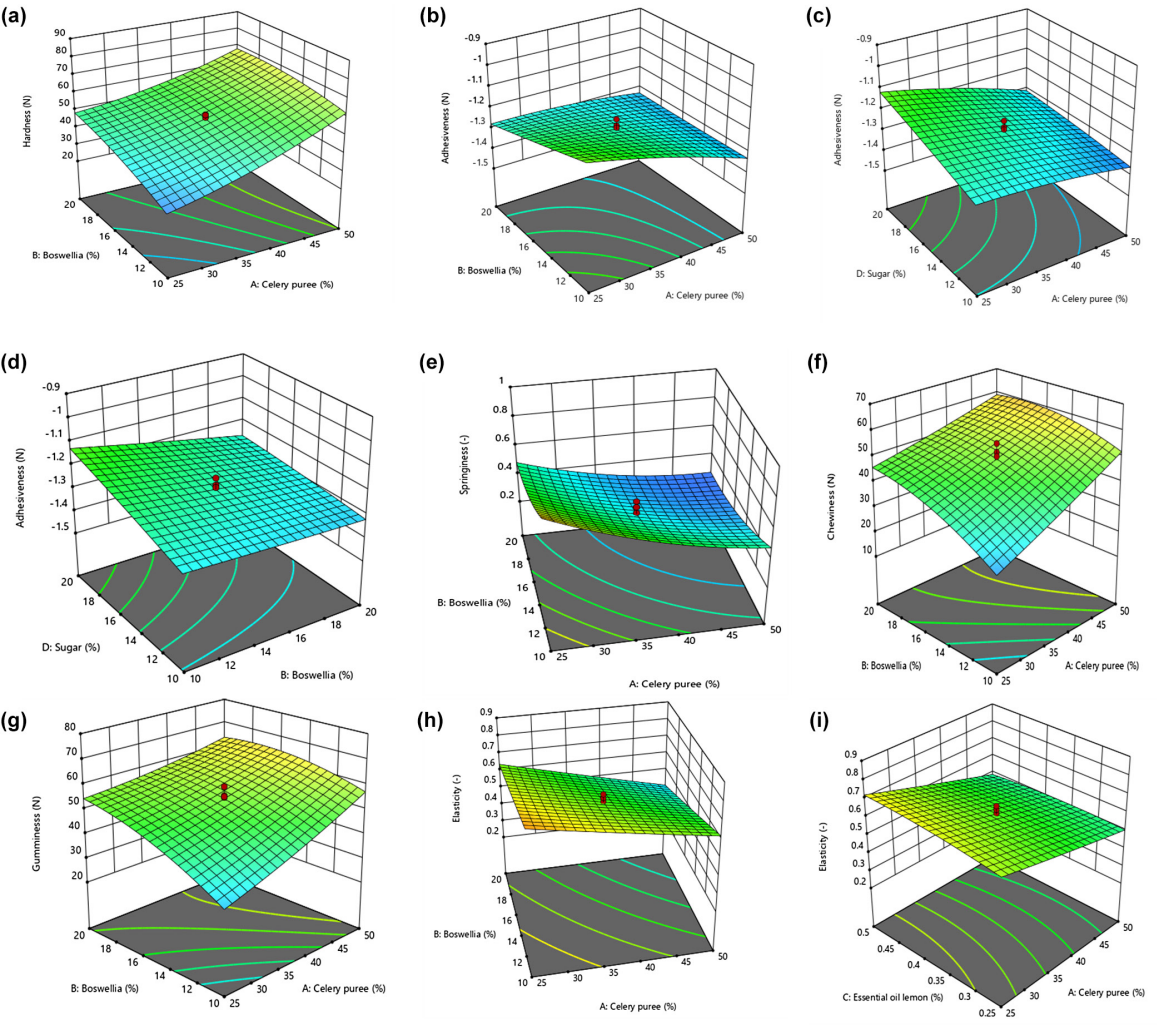
(a–i) Three‐dimensional (3D) response surface and optimizer indicating the significant (*p* < .05) interaction effect of factors such as A: celery puree (25%–50%), B: boswellia gum (10%–20%), C: lemon essential oil (0.25%–0.50%), and D: sugar (10%–20%) on gummy candy; (a): hardness; (b, c, and d): adhesiveness; (e): springiness; (f): chewiness; (g): gumminess; (h and i): elasticity.

Variables A and B with A and also D in linear and quadratic modes had a positive effect on hardness and the others indicated a negative influence. The results of ANOVA for fitting response levels are calculated *p*‐value was less than .0001, which illustrated the significance; thus, *R* ^2^ (.94) and $R_{\mathrm{adj}}^{2}$ (.89) confirmed that model was extremely prominent. The variables of A, B, and C in linear mode, A, B, C, and also D in quadratic mode, and interaction effect of AB demonstrated a significant impact on hardness of gummy candies at the 5% level. As can be seen from Figure 1a, the hardness was elevated by the addition of celery puree and boswellia gum. Adhesiveness factor indicates adhesion degree for samples to teeth (Azmoon et al., 2021); the highest −0.91 N and lowest −1.42 N values were reported for numbers 13 and 18, respectively. It had been obtained to predict adhesiveness of gummy candy samples (Equation 2):

(2) $$\begin{array}{c} \;\text{Adhesiveness}=–1.25\;–0.067A–0.042B+0.0146C+0.053D\\ \;+0.041\mathrm{AB}–0.0006\mathrm{AC}–0.028\mathrm{AD}–0.0006\mathrm{BC}\\ \;–0.0281\mathrm{BD}+0.0019\mathrm{CD}–0.0147A^{2}+0.0016\;B^{2}\\ \;+0.0091C^{2}–0.0047D^{2} \end{array}$$

Variables C and D in linear mode, interaction of AB and CD as well as B and C in quadratic mode had positive impact on adhesion and others illustrated negative effect. The obtained *p*‐value was less than .05, which represented significance; therefore, *R* ^2^ (.92) and $R_{\mathrm{adj}}^{2}$ (.86) confirmed that model was so important. The variables of A, B, and C in linear mode and also interaction of AB, AD, and BD had a significant effect (*p* < .05) on adhesiveness for gummy candy (Table 3). In Figure 1b, the adhesiveness was decreased with enhancement in concentration of celery puree and boswellia gum. Also, adhesiveness level for gummy candy samples was diminished by adding celery puree and sugar (Figure 1c). In Figure 1d, the adhesion of treated samples was declined using increase in boswellia gum and sugar concentrations.

Springiness refers to the deformation recovery rate in which a piece immediately returns to its disfigured state after the applied tension is removed (Azmoon et al., 2021). The highest (0.95) and lowest (0.21) springiness were reported for sample with numbers 17 and 23, respectively (Table 2), and had been attained to predict springiness of gummy candies (Equation 3):

(3) $$\begin{array}{c} \text{Springiness}=+0.40–0.143A–0.14B–0.0008C+0.045D\\ \;+0.035\mathrm{AB}+0.005\mathrm{AC}–0.005\mathrm{AD}+0.015\mathrm{BC}–0.0125\mathrm{BD}\\ \;+0.005\mathrm{CD}+0.0567A^{2}+0.0567\;B^{2}+0.050C^{2}–0.024D^{2} \end{array}$$

Variable D in linear mode, interaction effect of AB, AC, BC, and CD and also A, B, and C in quadratic mode had a direct impact on springiness and others outlined a reversed trend. According to ANOVA, *p*‐value calculated was less than .0001, which indicated the significance and *R* ^2^ (.96) and also $R_{\mathrm{adj}}^{2}$ (.92) confirmed that were extremely significant for model. Also, variables of A, B, and C in linear mode and all four variables in quadratic mode and interaction for AB had a significant effect on springiness (*p* < .05). Figure 1e depicts the interaction of A and B and at the same time as both variables increased; however, springiness reduced.

Table 3 demonstrated that the lowest chewiness value was obtained 19.81 N for test 13, and the highest was reported 62.32 N in number 4. It had been obtained to predict the chewiness of gummy candy samples (Equation 4):

(4) $$\begin{array}{c} \;\text{Chewiness}=+48.96+9.64A+6.56B–1.07C–3.49D\\ \;–2.97\mathrm{AB}–0.212\mathrm{AC}+0.566\mathrm{AD}–0.311\mathrm{BC}\\ \;+0.247\mathrm{BD}–1.32\mathrm{CD}+0.512A^{2}–3.71\;B^{2}\\ \;–3.66C^{2}+0.745D^{2} \end{array}$$

Variables A and B in linear mode, the interaction of AD and BD, and also A and D in quadratic mode have a positive effect on adhesion. The determined *p*‐value was lower than .0001, which illustrated the significance and *R* ^2^ (.93) and also $R_{\mathrm{adj}}^{2}$ (.87) exhibited that model was so significant. According to ANOVA, variables of A, B, and C in linear mode, interaction for AB and B, and also C in quadratic mode had a significant influence on chewiness of gummy candy (*p* < .05). In Figure 1f, simultaneously with increase in concentration of celery puree and boswellia gum, the chewiness was enhanced for gummy candy samples.

Based on Table 3, the lowest (23.93 N) and highest (75.86 N) amounts of gum were obtained for test numbers 13 and 18, respectively, as calculated in Equation 5:

(5) $$\begin{array}{c} \;\text{Gumminess}=+54.37+8.20A+6.86B–0.841C–3.89D\\ \;–3.69\mathrm{AB}–0.9137\mathrm{AC}+1.4\mathrm{AD}–0.676\mathrm{BC}+0.967\mathrm{BD}\\ \;–1.53\mathrm{CD}+1.25A^{2}–3.53\;B^{2}–3.05C^{2}+0.952D^{2} \end{array}$$

Variables A and B in linear mode, the interaction of AD and BD, and also A and D in quadratic mode have a positive effect on gumminess. According to ANOVA, *p*‐value was less than .0001, which indicated the significance, and *R* ^2^ (.94) and also $R_{\mathrm{adj}}^{2}$ (.86) outlined that model was very significant. The A, B, and C variables in linear mode, interaction for AB, B and C in quadratic mode illustrated a significant effect on gummy candy (*p* < .05). Figure 1g depicted that gumminess was improved for samples by adding celery puree and boswellia gum.

The lowest (0.29) and the highest (0.86) elasticity levels were obtained for tests 8 and 4 (Table 3), respectively, as determined in Equation 6:

(6) $$\begin{array}{c} \;\text{Elasticity}=+0.606–0.097A–0.088B+0.010C+0.055D–0.018\mathrm{AB}\\ \;–0.018\mathrm{AC}+0.003\mathrm{AD}–0.010\mathrm{BC}+0.002\mathrm{BD}–0.010\mathrm{CD}\\ \;–0.002A^{2}+0.002B^{2}+0.013C^{2}–0.01D^{2} \end{array}$$

Variables C and D in linear mode, AD and BD interactions, and also B and C in quadratic mode had a positive effect on elasticity. The calculated *p*‐value was less than .05, which indicated the significance and *R* ^2^ (.97) and also $R_{\mathrm{adj}}^{2}$ (.94) represented that model was extremely significant. The A, B, and C variables in linear mode, interaction for AB, AC, and C in quadratic mode demonstrated a significant effect on elasticity (*p* < .05), as obtained. In Figure 1h, elasticity of gummy candy samples was diminished by elevated celery puree and boswellia gum. Also, this feature was declined by adding concentration of celery puree and lemon essential oil in Figure 1i.

In general, the addition of celery puree and boswellia gum had improved hardness, chewiness, and gumminess; however, it reduced adhesion, springiness, and elasticity characteristics. Higher sugar and lemon essential oil had caused an increase in stickiness, springiness, and elasticity as well as a reduction in hardness, chewiness, and gumminess. The enhanced sugar had a significant effect (*p* < .05) on these factors, but due to lower lemon essential oil, these impacts were no significant (*p* > .05).

The gelling agent plays a role in texture of gummy candy and gelation mechanism, because it affects formation of hydrocolloid gel and binding zones (Javanmard et al., 2012; Renaldi et al., 2022). Addition of hydrocolloids content in formulation, a larger bound water and formula components are placed together in a denser structure and finally adhesiveness reduced (Fauziyah et al., 2023). In the present research, a direct relationship between gelatin and pectin with strength and hardness had been observed, which was agreed by previous researcher that higher pectin caused an enhancement in hardness and increasing sugar reduced this attribute (Čižauskaitė et al., 2019).

The hydrocolloid addition (gelatin, pectin, starch, sugar, or acid concentration) to produce a gel considerably reduced volatile release and gelatin leading greatest suppression; however, hardness had no significant effect on flavor (Zhang & Barringer, 2018). Texture assessment of ciku (*Manilkara zapota*) pastilles illustrated that formulations including 10% and 12% fruit puree indicated more cohesiveness, but no significant differences were observed between them, since all treated samples had similar framework after being compressed and flattened through moving probe (Hashim et al., 2021). Total dietary fiber and starch in celery were found to be 32.09% and 8.68%, respectively; therefore, these components generally elevated hardness of jellies and gummy candies and also on bread, adding celery powder reduced volume and hardness (Wang et al., 2020). In gummy candy of fruit puree prepared from *Garcinia atroviridis*, hardness 89.58–106 N, cohesion 0.45–0.55, adhesiveness 263.27–113.98 N.S, springiness 0.73–0.84, chewiness 32.7–49.37 N, and gumminess 42.42–58.79 N illustrated a significant difference (*p* < .05) by changing pectin 0.5%–1.5% and gelatin 5.5%–8.9% (Renaldi et al., 2022). Similar to results of this study, gummy candy prepared with gelatin and grape had higher textural characteristics, for instance, hardness, chewiness, and gumminess, but less adhesiveness and springiness were less because of sucrose compared to berries and carobs (Kurt et al., 2022). It had been reported that sucrose and soluble fiber improved springiness of gummy candy and also along with higher hardness for product, chewiness, and gumminess were enhanced (Gok et al., 2020). Gummy candies formulated with red beet extract, *Salix aegyptiaca* distillate and gellan gum demonstrated hardness improvement (60 N) and gumminess reduction (15 N) with an increment in gellan content (0.5% or 1.5%) toward gelatin (Moghaddas Kia et al., 2020). It had been found that choice of sweeteners in confectionery or dough making could affect the viscosity and other textural attributes of products (Zhang & Barringer, 2018). Starch increased gel strength and hardness of gelatin‐based gummy sweets; therefore, different sources and amounts affected textures such as higher cassava starch (16%) had led to pastilles with almost twice hardness and chewability (Pereira et al., 2022). In production of pastilles with Jaban watermelon exocarp powder, the viscosity and hardness of pastilles at higher levels improved from 0.457 to 1.55 P and 1667 to 7232 g, respectively (Tireki et al., 2023).

### 3.2 Physicochemical frameworks of gummy candies

The actual and predicted results related to optimization of influencing factors on physicochemical attributes for gummy candy samples are presented in Tables 4 and 5, and also predicted and actual values are extremely close and consistent with each other. The minimum and maximum water activities for sample numbers 16 (0.692) and 5 (0.822) had been obtained, respectively (Equation 7):

(7) $$\begin{array}{c} \;\text{Water activity}=0.741–0.0151A–0.016B–0.003C–0.018D\\ \;–0.0012\mathrm{AB}–0.0012\mathrm{AC}+0.005\mathrm{AD}+0.0007\mathrm{BC}\\ \;+0.0089\mathrm{BD}+0.0009\mathrm{CD}–0.0006A^{2}+0.0009B^{2}\\ \;–0.0006C^{2}–0.0004D^{2} \end{array}$$

**FIGURE 2:**
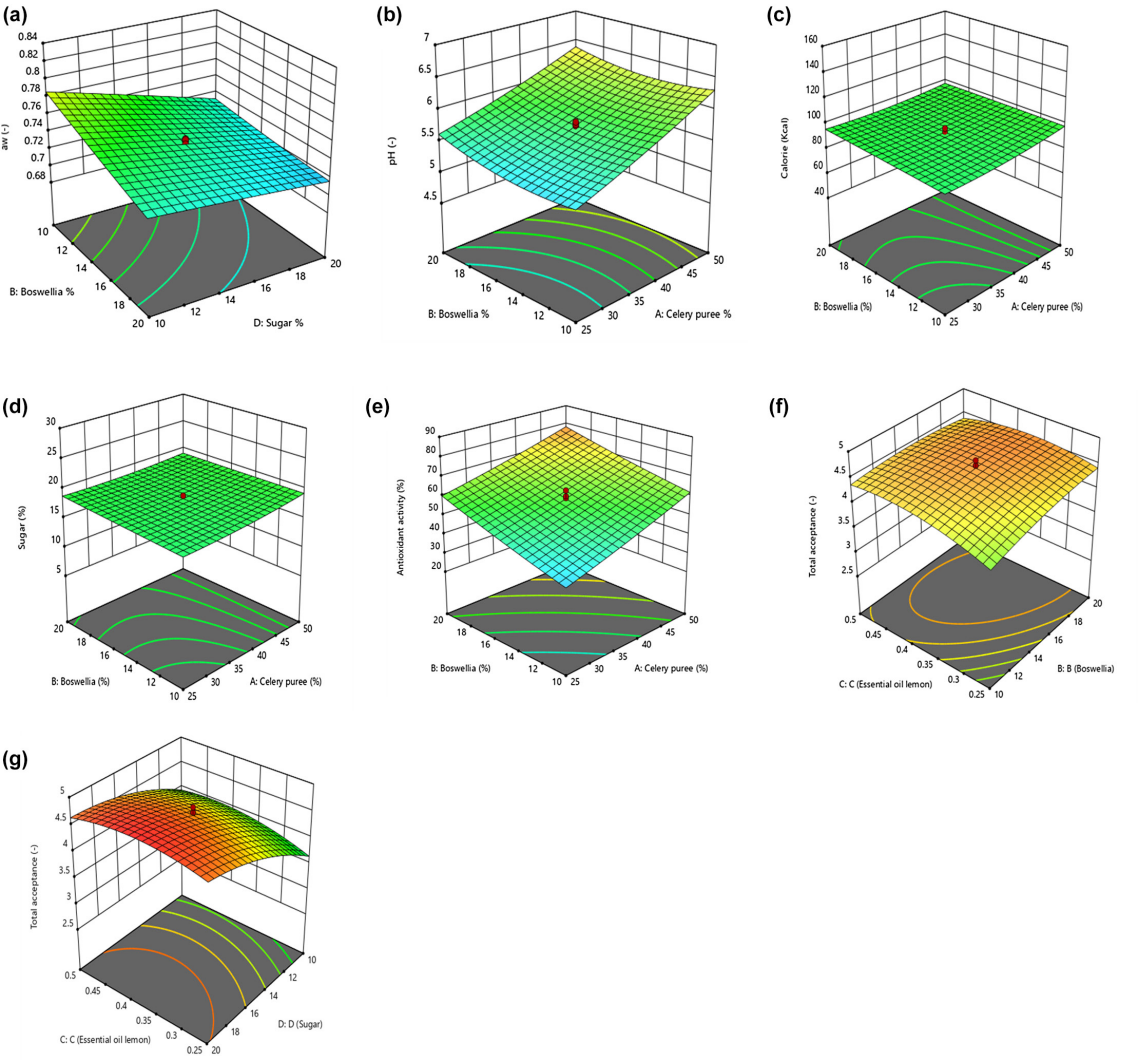
(a–g) Three‐dimensional (3D) surface and response optimizer showing significant (*p* < .05) interaction effect of factors, A: celery puree (25%–50%), B: boswellia gum (10%–20%), C: lemon essential oil (0.25%–0.50%), and D: sugar (10%–20%) on gummy candy; (a): water activity; (b): pH; (c): calorie; (d): sugar; (e): antioxidant function; (f and g): total acceptance.

In equation, interaction of AD, BC, BD, CD, and B in quadratic state had a positive effect on water activity. The calculated *p*‐value was less than .0001, which indicated the significance and *R* ^2^ (.95) and also $R_{\mathrm{adj}}^{2}$ (.91) depicted that model was very significant. Regarding ANOVA (Table 4), variables of A, B, and C in linear mode and interaction for BD displayed a significant effect on water activity in gummy candy (*p* < .05). Figure 2a outlines that water activity was elevated using higher percentages of sugar and boswellia gum in samples.

**TABLE 4:** Central composite design with actual and predicted values of water activity, pH, and calorie responses based on independent variables.

Run	Independent variables (%)				Dependent variables (responses)					
	A	B	C	D	Water activity (*a*_w_)		pH		Calorie (Kcal)	
					Actual value	Predicted value	Actual value	Predicted value	Actual value	Predicted value
1	25.0	10	0.250	10	0.822	0.819	5.42	5.37	66.74	61.96
2	50.0	10	0.250	10	0.784	0.780	6.35	6.42	74.84	72.08
3	25.0	20	0.250	10	0.755	0.762	5.76	5.69	71.43	69.76
4	50.0	20	0.250	10	0.721	0.718	6.48	6.55	76.24	73.27
5	25.0	10	0.500	10	0.811	0.810	5.28	5.22	67.46	63.88
6	50.0	10	0.500	10	0.762	0.766	6.25	6.29	74.86	72.86
7	25.0	20	0.500	10	0.752	0.754	5.63	5.55	70.89	67.66
8	50.0	20	0.500	10	0.713	0.706	6.37	6.42	73.46	70.02
9	25.0	10	0.250	20	0.743	0.747	5.49	5.44	115.45	119.55
10	50.0	10	0.250	20	0.732	0.729	6.38	6.47	126.48	129.20
11	25.0	20	0.250	20	0.729	0.725	5.79	5.76	127.3	128.79
12	50.0	20	0.250	20	0.704	0.702	6.55	6.60	127.58	131.82
13	25.0	10	0.500	20	0.738	0.741	5.36	5.31	118.24	120.70
14	50.0	10	0.500	20	0.728	0.718	6.29	6.36	126.87	129.20
15	25.0	20	0.500	20	0.721	0.721	5.72	5.64	122.5	125.92
16	50.0	20	0.500	20	0.692	0.694	6.42	6.49	123.54	127.81
17	25.0	15	0.375	15	0.765	0.753	4.89	5.32	94.44	96.25
18	50.0	15	0.375	15	0.701	0.720	6.83	6.28	104.64	102.26
19	37.5	10	0.375	15	0.757	0.763	6.12	6.02	87.53	89.06
20	37.5	20	0.375	15	0.721	0.722	6.27	6.25	94.36	92.26
21	37.5	15	0.250	15	0.737	0.741	5.96	5.84	92.24	91.88
22	37.5	15	0.500	15	0.729	0.732	5.71	5.71	91.05	90.83
23	37.5	15	0.375	10	0.758	0.759	5.65	5.64	44.34	68.77
24	37.5	15	0.375	20	0.709	0.716	5.82	5.71	151.46	126.45
25	37.5	15	0.375	15	0.741	0.737	5.63	5.80	92.67	93.70
26	37.5	15	0.375	15	0.745	0.737	5.74	5.80	92.73	93.70
27	37.5	15	0.375	15	0.748	0.737	5.8	5.80	92.81	93.70
28	37.5	15	0.375	15	0.747	0.737	5.75	5.80	93.56	93.70
29	37.5	15	0.375	15	0.742	0.737	5.77	5.80	92.34	93.70
30	37.5	15	0.375	15	0.727	0.737	5.81	5.80	96.36	93.70

**TABLE 5:** Central composite design with actual and predicted values of sugar, antioxidant activity, and total acceptance responses based on independent variables.

Run	Independent variables (%)				Dependent variables (responses)					
	A	B	C	D	Sugar (g/100 g)		Antioxidant activity (mg GA/100 g)		Total acceptance	
					Actual value	Predicted value	Actual value	Predicted value	Actual value	Predicted value
1	25.0	10	0.250	10	13.01	12.16	35.13	34.45	3.21	3.03
2	50.0	10	0.250	10	14.75	14.19	65.41	62.61	3.42	3.30
3	25.0	20	0.250	10	14.11	13.80	63.39	59.50	3.51	3.42
4	50.0	20	0.250	10	15.23	14.52	75.10	83.40	3.73	3.57
5	25.0	10	0.500	10	13.25	12.63	53.24	49.89	3.65	3.52
6	50.0	10	0.500	10	14.79	14.39	64.36	68.29	3.87	3.77
7	25.0	20	0.500	10	14.15	13.33	66.48	68.68	3.68	3.54
8	50.0	20	0.500	10	14.25	13.79	85.58	82.83	3.83	3.66
9	25.0	10	0.250	20	23.12	23.67	29.25	29.72	3.82	3.90
10	50.0	10	0.250	20	24.71	25.47	60.47	59.12	4.35	4.25
11	25.0	20	0.250	20	25.18	25.52	59.34	56.51	4.69	4.52
12	50.0	20	0.250	20	25.3	26.01	80.43	81.66	4.71	4.74
13	25.0	10	0.500	20	23.34	23.99	52.19	45.40	4.14	4.12
14	50.0	10	0.500	20	25.13	25.53	63.36	65.05	4.45	4.44
15	25.0	20	0.500	20	24.26	24.91	65.23	65.94	4.34	4.36
16	50.0	20	0.500	20	24.35	25.14	79.16	81.34	4.51	4.56
17	25.0	15	0.375	15	18.19	18.60	37.39	48.92	3.77	3.95
18	50.0	15	0.375	15	20.25	19.73	84.25	70.70	3.95	4.18
19	37.5	10	0.375	15	17.57	17.65	43.39	49.47	4.14	4.34
20	37.5	20	0.375	15	18.45	18.27	78.29	70.14	4.34	4.59
21	37.5	15	0.250	15	18.15	18.23	60.16	59.03	3.65	4.04
22	37.5	15	0.500	15	18.21	18.03	67.65	66.58	4.18	4.19
23	37.5	15	0.375	10	8.85	13.58	63.33	59.36	2.93	3.67
24	37.5	15	0.375	20	29.84	25.01	54.23	56.25	4.86	4.56
25	37.5	15	0.375	15	18.35	18.41	58.47	59.36	4.67	4.28
26	37.5	15	0.375	15	18.23	18.41	59.17	59.36	4.53	4.28
27	37.5	15	0.375	15	18.2	18.41	55.47	59.36	4.45	4.28
28	37.5	15	0.375	15	18.27	18.41	60.06	59.36	4.57	4.28
29	37.5	15	0.375	15	18.25	18.41	63.18	59.36	4.63	4.28
30	37.5	15	0.375	15	18.85	18.41	57.98	59.36	4.76	4.28

The evaluation result of ciku fruit (*Manilkara zapota*) pastilles exhibited that all samples had water activity values less than 0.85 (Hashim et al., 2021). The final water activity value of most jellies, jams, and sweet fruits was usually less than 0.85 (Gok et al., 2020). Many hydrocolloids are capable of forming hydrogels including a network of interconnected biopolymer molecules that trap water through capillary forces (McClements, 2021). If hydrocolloids improve, the binding intensity of water molecules will enhance and ultimately cause water activity for samples to decrease (Din et al., 2022). Pectin gel consists of a three‐dimensional network that holds water, sugar, and other soluble substances, which are hydrogen and hydrophobic connections (Piazza et al., 2009). The effect of starch concentration on water activity may be due to softening impact of water in food polymers, which are used as gelling agents in gummy candies (Tireki et al., 2023). In this study, owing to more presence of pectin as hydrocolloid in celery puree and boswellia gum, a reduction in aqueous activity was observed for samples. Also, more sugar led to a decrease in water activity for samples because this component bonded water in gel mixtures, thereby reducing synergism of gels and lubrication effect by free water (Moghaddas Kia et al., 2020). Water activities between 0.72 and 0.73 were obtained in gummy candies prepared with lycopene and watermelon peel (Din et al., 2022), which was in line with present research. The water activity of pastille samples had been reported as 0.76 to 0.87, which had an inverse correlation with sugar content (Gok et al., 2020).

The pH levels were reported at 6.83 and 4.89 values for sample number 18 (Table 4) and measured by Equation 8:

(8) $$\begin{array}{c} \;\mathrm{pH}=5.75+0.438A+0.091B–0.058C+0.034D–0.05\mathrm{AB}\\ \;+0.0025\mathrm{AC}–0.005\mathrm{AD}+0.0013\mathrm{BC}+0.0013\mathrm{BD}\\ \;+0.0038\mathrm{CD}+0.038A^{2}+0.122B^{2}+0.032C^{2}+0.0\;07D^{2} \end{array}$$

The variable C in linear mode and AB interaction showed a negative effect on pH and also *p*‐value was less than .0001, which represented the significance; therefore, *R* ^2^ (.98) and $R_{\mathrm{adj}}^{2}$ (.96) illustrated that model was extremely significant. A, B, and C variables in linear mode, interaction of AB, and A, B, and also C in quadratic mode outlined a significant impact on pH for gummy candy (*p* < .05) based on ANOVA. Figure 2b depicts the interaction influence of celery puree and boswellia gum on this feature, and also pH level was improved by more concentration.

Food acids are added to gummy candies to get a spicy and sour taste, and also gel formation by gelling agents requires a low pH (Paternina et al., 2022). In the present study, the range of this feature from 5.42 to 6.55 with an optimal value of 6.07 was detected for gummy candies owing to alkaline range (pH = 12) in celery puree compared to common examples in market. The pH levels of gummy candies were reported 3.35–3.39 and 3–5; there was a direct relationship between water activity and also this trait; therefore, water has a neutral nature and improves pH of product (Tireki et al., 2023).

As illustrated in Table 4, the minimum and maximum calories for sample numbers 1 (66.74 Kcal) and 4 (151.46 Kcal) had been determined, respectively (Equation 9):

(9) $$\begin{array}{c} \;\text{Calorie}=93.41+2.68A+1.49B–0.442C+26.10D–1.65\mathrm{AB}\\ \;–0.286\mathrm{AC}–0.118\mathrm{AD}–1.00\mathrm{BC}+0.360\mathrm{BD}–0.192\mathrm{CD}\\ \;+1.99A^{2}–0.160B^{2}+0.014C^{2}+1.58D^{2} \end{array}$$

The variables of A, B, and D in linear mode, BD, and also A interaction, C, and D in quadratic mode exhibited a positive effect on calories. The *p*‐value was less than .0001, which demonstrated the significance and *R* ^2^ (.99) and also $R_{\mathrm{adj}}^{2}$ (.99) confirmed that model was very significant. Regarding ANOVA, A, B, and C variables in linear mode, interaction effect for AB and A and also D in quadratic mode represented a significant effect on calories of gummy candy (*p* < .05). Figure 2c illustrated that calories were increased by the addition of celery puree and boswellia gum percentages in samples.

The results for Table 4 depicted that final calorie content is significantly reduced, which indicates the manufacture of a low‐calorie gummy candy. The same as present results, two types of gummy candies containing orange, fruit juice, and slightly sweetened with honey and also mixed berry puree had 73.8 and 39.8 Kcal per 100 g gummy candy, respectively, which were 5 and 9 times less than similar commercial products (Teixeira‐Lemos et al., 2021).

In a study conducted on production of gummy candy using *Averrhoa bilimbi* fruit puree, sorbitol, and sugar syrup with gum arabic (0%, 4%, 8%, 12%, and 16%) and also gelatin, resulted sample had 374 Kcal energy per 100 g (Zainol et al., 2020); in contrast to present study, that optimal gummy candy indicated 70 Kcal energy per 100 g, which was approximately five times lower. A vegan, sugar‐free, and low‐calorie gummy candy using a polysaccharide called furcellaran derived from algae had acceptable results (Stępień et al., 2023), which was in line with present research.

The amount of 29.84% sugar as the maximum level for sample number 24 and minimum level of 13.01% for sample 1 were achieved (Table 5); so, based on statistical analysis, it was reported in Equation 10:

(10) $$\begin{array}{c} \;\text{Sugar}\%=18.36+0.508A+0.270B–0.073C+5.16D\\ \;–0.327\mathrm{AB}–0.065\mathrm{AC}–0.005\mathrm{AD}–0.233\mathrm{BC}\\ \;+0.053\mathrm{BD}–0.035\mathrm{CD}+0.318A^{2}+0.016B^{2}\\ \;+0.058C^{2}+0.350D^{2} \end{array}$$

Variables of A, B, and D in linear mode, BD, and also A interaction, B, C, and D in quadratic mode exhibited a positive effect on sugar. Represented that calculated *p*‐value was less than .0001, which indicated the significance and *R* ^2^ (.99) and also $R_{\mathrm{adj}}^{2}$ (.99) expressed that model was so significant. The A, B, and D variables in linear mode, interaction of AB, and also A and D in quadratic mode had a significant impact on sugar for gummy candies (*p* < .05), as obtained. Figure 2d proves the graph related to interaction of celery puree and boswellia gum on sugar for gummy candy, which was enhanced slightly by higher concentrations.

Maltitol syrup instead of glucose syrup indicated that using wheat‐soluble fiber as an alternative to reduce sucrose for pastilles resulted in meeting consumer demands and expectations, while maintaining adequate physicochemical function (Gok et al., 2020). Low‐sugar percentage gummy candy exhibited favorable textural characteristics and high nutritional value, making it a competitive option compared to commercial samples (Stępień et al., 2023). Regarding obtained results, these studies highlighted the potential of low‐sugar gummy candy enriched with beneficial ingredients to increase demand for sugar‐free and healthier food options.

The minimum and maximum antioxidant activities were obtained for sample numbers 9 (29%) and 18 (84%) of gummy candy, respectively (Table 5), and had been obtained to predict (Equation 11):

(11) $$\begin{array}{c} \;\text{Antioxidant activity}\%=57.67+10.12A+9.21B+3.13C–1.54D\\ \;–1.06\mathrm{AB}–2.44\mathrm{AC}+0.312\mathrm{AD}–1.56\mathrm{BC}\\ \;+0.437\mathrm{BD}+0.0625\mathrm{CD}+0.677A^{2}+0.677B^{2}\\ \;+1.43C^{2}+0.177D^{2} \end{array}$$

The variables of A, B, and C in linear mode, AD, BD, and CD interactions, and A, B, C, and D in quadratic mode outlined a positive effect on antioxidant activity. Regarding the results, measured *p*‐value was less than .0001, which illustrated the significance and *R* ^2^ (.95) and also $R_{\mathrm{adj}}^{2}$ (.90) presented that model was extremely significant. The variables of A, B, and C demonstrated a significant effect on antioxidant attribute for samples (*p* < .05). Based on Figure 1e, this feature was improved slightly with increase in percentages of celery puree percentage and boswellia gum, which was not significant.

Celery puree, boswellia gum, and lemon essential oil were rich in antioxidants and also had significantly increased antioxidant activity in gummy candy samples. In previous research, antimicrobial, insecticidal, and antioxidant attributes of boswellia had been investigated (Borotová et al., 2023). Boswellic acids are a group of pentacyclic triterpenoids and the main component for their resin (Hamidpour et al., 2013), and celery is rich in carotene, vitamin C, and phenolics, which both exhibit antioxidant activity (Wang et al., 2020). The highest percentage of free radical scavenging activity was represented in 3% lemon peel essential oil with edible skin coating materials on pear about 78.18% followed by treatments including 1%, 1.5%, 2%, and 2.5%, which were close to 65.84%, 68.59%, 71.85%, and also 75.00%, respectively (Iftikhar et al., 2022). In study of bread enriched with celery powder, antioxidant significantly improved the phenolic content for whole bread (Wang et al., 2020). Treated gummy candies with red beet extract, *Salix aegyptiaca* distillate, and gellan gum displayed that radical scavenging activity was improved remarkably using 0.1% or 0.3% red beet extract (Moghaddas Kia et al., 2020). Considerable bioactive components and antioxidant features (DPPH: 0.29 to 0.30, ABTS: 0.45 to 0.47, and FRAP: 0.39 to 0.41 mg ascorbic acid equivalent/g dried sample) were found in gummy candy of fruit puree produced from *Garcinia atroviridis*, which supplied health benefits (Renaldi et al., 2022). The positive effect was detected on antioxidant parameters in jellies containing addition of soy protein isolate (Stępień et al., 2023). The low‐sugar pastille enriched with 5% spirulina biomass showed a significantly improved yield and antioxidant activity compared to control containing sugar and without algae (Paternina et al., 2022), which was in line with the present study.

Regarding Table 5, the lowest score was attained for sample number 23 (2.9) and the highest in sample number 24 (4.8), as calculated by Equation 12:

(12) $$\begin{array}{c} \;\text{Total acceptance}=4.55+0.095A+0.104B+0.079C–0.412D\\ \;–0.031\mathrm{AB}–0.006\mathrm{AC}+0.019\mathrm{AD}–0.093\mathrm{BC}\\ \;+0.056\mathrm{BD}–0.068\mathrm{CD}–0.182A^{2}–0.082B^{2}\\ \;+0.169C^{2}–0.17\;0D^{2} \end{array}$$

Regarding the equation, A, B, and C variables in the linear mode, interaction effect of AD, and also BD and C in quadratic mode had a positive effect on total acceptance of gummy candies. It presented that *p*‐value was less than .0001, which exhibited significance and *R* ^2^ (.96) and also $R_{\mathrm{adj}}^{2}$ (.93) confirmed that model was so significant. The variables of A, B, C, and D in a linear mode and also BC and CD interactions exhibited a significant effect on total acceptance for samples. In Figure 2f, total acceptance rate had enhanced slightly by adding boswellia gum and lemon essential oil to gummy candy sample. However, in Figure 2g, at the same time as lemon essential oil and sugar elevate, the points were developed significantly for samples.

Based on obtained results, samples containing more sugar got more points owing to significant effect on acceptability of product; but at optimal point, score obtained by sample was more than 4, which was acceptable in sensory evaluation and in this condition, sugar was 17.18 g for formulation of gummy candies. Therefore, the use of celery puree and boswellia gum in low‐calorie gummy candy formulation could be an acceptable substitute for sugar and a product with desirable sensory characteristics. Organoleptic results of gummy candy with licorice plant (0.1%, 0.5%, and 1%) and agar (1%, 2%, and 3%) showed that sensory properties were satisfied (Basiri, 2020). Another research observed that pastille containing 50% flesh puree: 50% rind puree watermelon had the highest likeness in terms of overall acceptance; conversely, pastille including 30% flesh puree: 70% rind puree watermelon had the lowest likeness (Din et al., 2022). The sample of *Garcia Atroviridis* gummy candy made with optimized formula including pectin (0.5%–1.5%) and gelatin (8.5%–9.5%) had an average score (6.9–1.7) in sensory evaluation (Renaldi et al., 2022). Sensory score for molasses gummy candies was achieved higher than 3.0 on a 5‐point hedonic scale with natural sugar (Kurt et al., 2022). The acceptability parameter was satisfactory for gummy candies including 5% *Spirulina* (78.7%) and 3% freeze‐dried açai (83.8%) pulp (Paternina et al., 2022).

### 3.3 Optimization of variables based on RSM design

**FIGURE 3:**
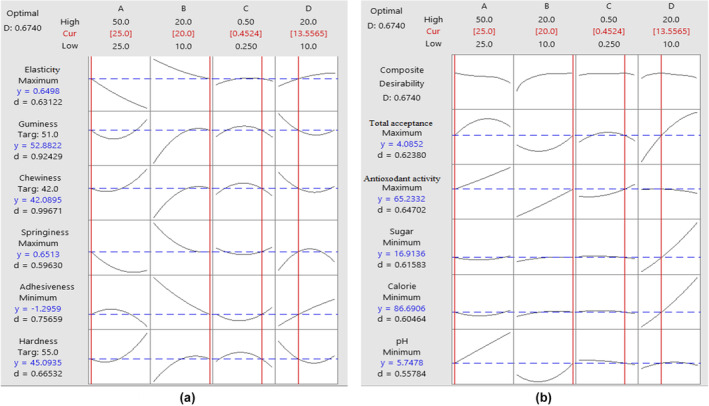
(a,b) Graphs for optimal points of independent and dependent variables related to (+) texture and (++) physicochemical characteristics.

The purpose of optimizing response level is to find optimal points in the design space, which are examined in Figure 3 and indicated that actual calculated levels are not much different from predicted values. The optimal formulation provided by RSM for production of gummy candy included 25% celery puree, 20% boswellia gum, 0.450% lemon essential oil, and 17.18% sugar.

## 4 CONCLUSION

Nowadays, the consumer demand has accelerated for balanced, healthy, and nutritious food products. In the present research, celery puree, boswellia gum, and lemon essential oil were applied to produce functional and vegan gummy candies with low calories. The results outlined that the addition of celery puree and boswellia gum enhanced the firmness, chewiness, and also gumminess. On the other hand, declined stickiness, springiness, and elasticity attributes as well as retention capacity and pH were enhanced by water activity. Antioxidant function was significantly increased in treated samples with more celery puree, boswellia gum, lemon essential oil, and also optimal point indicated an acceptable level in sensory evaluation. According to formulation, produced gummy candy in addition to having taste, texture, high nutritional value, and technological features demonstrated few calories compared to similar samples in market.

## AUTHOR CONTRIBUTIONS

**Saba Ghodsi:** Conceptualization (equal); data curation (equal); formal analysis (equal); investigation (equal); methodology (equal); software (equal); validation (equal). **Marjan Nouri:** Funding acquisition (equal); project administration (equal); resources (equal); supervision (equal); visualization (equal); writing – original draft (equal); writing – review and editing (equal).

## CONFLICT OF INTEREST STATEMENT

No conflicts of interest were found by the authors.

## Data Availability

The data will be available from the corresponding authors.
